# Supplementary material for: Modified Lactoperoxidase System as a Promising Anticaries Agent: In Vitro Studies on Streptococcus mutans Biofilms
Source: Int J Mol Sci. 2023 Jul 28;24(15):12136. doi: 10.3390/ijms241512136 (PMC10418824; doi:10.3390/ijms241512136)
Supplement: Supplementary file 1 [file ijms-24-12136-s001.zip › ijms-2505676-supplementary.pdf]

Table S1. Influence of all tested LPO system modifications on *S. mutans* total biofilm biomass and total insoluble polysaccharide mass. Values are expressed as fraction of control sample (which is equal to 1) to eliminate variations observed among different plates. Control sample consisted of biofilm treated only with PBS. Asterisks indicate a statistically significant difference between a tested group and the control in the post hoc Tukey's test—* $p < 0.05$; ** $p < 0.01$; *** $p < 0.001$.

Group		Total biomass [relative to control] ± SD	Insoluble polysaccharide mass [relative to control] ± SD
System	H2O2 concentration [μM]		
LPO + SCN^-	250	0.7618** ± 0.0929	1.0594 ± 0.0527
	500	0.7959** ± 0.0899	1.0562 ± 0.1100
	1000	0.8186 ± 0.0996	1.1308 ± 0.0802
LPO + SCN^- + I-	250	0.8618 ± 0.0839	0.9321 ± 0.0824
	500	0.7343*** ± 0.0408	0.9179 ±0.0873
	1000	0.8722 ± 0.1139	0.7810 ± 0.1659
LPO + $SeCN^-$	250	0.6935*** ± 0.0167	0.5275*** ± 0.1117
	500	0.6206*** ± 0.0443	0.2907*** ±0.0810
	1000	0.3054*** ± 0.0431	0.1833*** ± 0.0446
LPO + I^-	250	0.1557*** ± 0.0206	0.1879*** ± 0.0400
	500	0.1499*** ± 0.0836	0.1440*** ± 0.0173
	1000	0.1392*** ± 0.0271	0.1441*** ± 0.0192
H2O2 toxicity control	250	0.7639* ± 0.1276	1.006 ± 0.0411
	500	0.7872* ± 0.0736	0.9513 ±0.0684
	1000	0.7792 ± 0.2045	0.9856 ± 0.0921
Control	-	1.0000 ± 0.1197	1.0000 ± 0.0504

Table S2. Area under kinetic curves (relative to control) of lactate production and glucose/sucrose consumption (biofilm 2 h). In the case of lactate, lower indicates on a slowed down production of lactate in tested system. In the case of glucose/sucrose, higher values indicates reduced consumption of glucose/sucrose by biofilm. Asterisks indicate a statistically significant difference between a tested group and the control in the post hoc Tukey's test—** $p < 0.01$; *** $p < 0.001$.

**System**	**H2O2 concentration [μM]**	**Lactate [AUC relative to control]**	**Glucose [AUC relative to control]**	**Sucrose [AUC relative to control]**
LPO + SCN^-	250	0.999 ± 0.068	0.985 ± 0.025	1.025 ± 0.025
	500	1.062 ± 0.043	0.998 ± 0.002	0.995 ± 0.018
	1000	1.059 ± 0.042	1.010 ± 0.024	0.945 ± 0.017
LPO + SCN^- + I^-	250	1.090 ± 0.015	1.012 ± 0.042	0.933 ± 0.025
	500	0.987 ± 0.011	0.963 ± 0.017	1.056 ± 0.035
	1000	1.040 ± 0.053	0.988 ± 0.013	1.100 ± 0.113
LPO + $SeCN^-$	250	0.788* ± 0.089	0.959 ± 0.020	1.086 ± 0.035

	500	0.625*** ± 0.039	0.981 ± 0.028	1.050 ± 0.024
	1000	0.554*** ± 0.053	0.998 ± 0.020	1.082 ± 0.048
LPO + I^-	250	0.000*** ± 0.000	0.950 ± 0.018	1.113 ± 0.049
	500	0.000*** ± 0.000	0.972 ± 0.003	1.224*** ± 0.089
	1000	0.000*** ± 0.000	0.979 ± 0.012	1.284*** ± 0.082
SCN^-	-	1.005 ± 0.025	1.011 ± 0.018	1.005 ± 0.045
SCN^- + I^-	-	1.021 ± 0.011	1.001 ± 0.041	1.084 ± 0.022
$SeCN^-$	-	1.001 ± 0.035	1.054 ± 0.036	1.065 ± 0.046
I^-	-	1.015 ± 0.022	1.022 ± 0.048	1.038 ± 0.045
H_2O_2	250	0.959 ± 0.029	1.005 ± 0.024	1.050 ± 0.026
	500	0.962 ± 0.049	1.006 ± 0.022	1.016 ± 0.006
	1000	0.975 ± 0.085	1.011 ± 0.042	1.022 ± 0.010
Control	-	1.000 ± 0.033	1.000 ± 0.016	1.000 ± 0.024

Table S3. Area under kinetic curves (relative to control) of lactate production and glucose/sucrose consumption (biofilm 24 h). In the case of lactate, lower indicates on a slowed down production of lactate in tested system. In the case of glucose/sucrose, higher values indicates reduced consumption of glucose/sucrose by biofilm. Asterisks indicate a statistically significant difference between a tested group and the control in the post hoc Tukey's test—** $p < 0.01$; *** $p < 0.001$.

System	H2O2 concentration [μM]	Lactate [AUC relative to control]	Glucose [AUC relative to control]	Sucrose [AUC relative to control]
LPO + SCN^-	250	0.804*** ± 0.049	1.038 ± 0.018	1.151 ± 0.018
	500	0.677*** ± 0.029	1.019 ± 0.015	1.139 ± 0.009
	1000	0.682*** ± 0.053	1.047 ± 0.017	1.158 ± 0.016
LPO + SCN^- + I^-	250	0.957 ± 0.072	0.969 ± 0.009	1.023 ± 0.024
	500	0.767* ± 0.008	0.963 ± 0.012	1.181 ± 0.006
	1000	0.712* ± 0.045	0.963 ± 0.011	1.135 ± 0.057
LPO + $SeCN^-$	250	0.627*** ± 0.050	0.953 ± 0.014	1.087 ± 0.031
	500	0.471*** ± 0.053	0.968 ± 0.010	1.075 ± 0.026
	1000	0.312*** ± 0.025	0.980 ± 0.012	1.082 ± 0.042
LPO + I^-	250	0.000*** ± 0.000	0.969 ± 0.009	1.346*** ± 0.030
	500	0.000*** ± 0.000	0.063 ± 0.012	1.343*** ± 0.035
	1000	0.000*** ± 0.000	0.963 ± 0.011	1.310*** ± 0.033
SCN^-	-	0.989 ± 0.045	1.022 ± 0.015	1.011 ± 0.017
SCN^- + I^-	-	0.958 ± 0.069	0.984 ± 0.028	1.022 ± 0.011
$SeCN^-$	-	0.971 ± 0.024	0.965 ± 0.022	1.005 ± 0.009
I^-	-	0.948 ± 0.047	0.978 ± 0.019	1.031 ± 0.010
H_2O_2	250	0.849 ± 0.049	1.008 ± 0.019	1.010 ± 0.016
	500	0.902 ± 0.029	1.011 ± 0.035	1.026 ± 0.009
	1000	1.072 ± 0.075	1.035 ± 0.021	1.060 ± 0.020
Control	-	1.000 ± 0.050	1.000 ± 0.019	1.000 ± 0.027

Table S4. Amounts of NAD+, NADH in the well and NAD+/NADH ratio after treatment biofilm with all the tested systems (1000 μM H2O2). Asterisks indicate a statistically significant difference between a tested group and the control in the post hoc Tukey's test—** $p < 0.01$; *** $p < 0.001$.

System	NADH [μg/well]	NAD [μg/well]	NADH/NAD
LPO + SCN^-	2.32 ± 0.13	3.14 ± 0.01	0.739 ± 0.039
LPO + SCN^- + I^-	3.29 ± 0.41	3.65 ± 0.57	0.918 ± 0.191
LPO + $SeCN^-$	3.50 ± 0.15	4.34*** ± 0.94	0.830 ± 0.162
LPO + I^-	6.40*** ± 0.07	0.78** ± 0.01	8.220*** ± 0.012
SCN^-	3.42 ± 0.20	2.51 ± 0.33	1.224 ± 0.312
SCN^- + I^-	3.55 ± 0.14	2.74 ± 0.18	1.051 ± 0.147
$SeCN^-$	3.22 ± 0.22	2.42 ± 0.08	1.007 ± 0.101
I^-	3.81 ± 0.13	2.54 ± 0.74	1.288 ± 0.207
H_2O_2	3.64 ± 0.56	2.58 ± 0.59	1.460 ± 0.436
Control	3.36 ± 0.56	2.29 ± 0.19	1.470 ± 0.231

Table S5. PTS system activity in S. mutans cells treated with tested LPO system modifications and control sample. Asterisks indicate a statistically significant difference between a tested group and the control in the post hoc Tukey's test—* $p < 0.05$, ** $p < 0.01$.

System	PTS activity [nM NADH/s]
LPO + SCN^-	3.52* ± 2.33
LPO + SCN^- + I^-	1.89** ± 1.67
LPO + $SeCN^-$	3.13** ± 1.85
LPO + I^-	3.12** ± 2.59
SCN^-	10.18 ± 3.35
SCN^- + I^-	18.22 ± 6.87
$SeCN^-$	11.41 ± 4.28
I^-	10.17 ± 4.91
Control	12.39 ± 5.25
